# Ocular Trauma from the “Knockout Game”

**DOI:** 10.1155/2014/285942

**Published:** 2014-07-13

**Authors:** Brian C. Joondeph

**Affiliations:** Colorado Retina Associates, PC, 8101 E Lowry Boulevard, Suite 210, Denver, CO 80230, USA

## Abstract

The “knockout game” is a new form of urban violence receiving much attention in local and national media. Apart from the obvious head trauma, eye injuries may be subtle and overlooked. This report brings awareness of potential eye damage with this type of assault. This report is of a young woman, victim of the knockout game, who sustained a submacular hemorrhage. Beyond a neurologic evaluation for anyone knocked unconscious following the knockout game, patients should be counseled regarding potential ocular injury and encouraged to seek eye care promptly should symptoms develop.

## 1. Introduction

The “knockout game” is a new form of urban assault where the assailant attempts to knockout an unsuspecting victim with a single punch for the amusement of the assailant and their accomplices [1]. Injuries and even death have occurred due to this activity. Controversy exists as to whether the cause is racism, anti-Semitism, or simply random violence. Nevertheless, this activity continues to occur in major urban environments. To my knowledge, this is the first report of significant ocular injury associated with this new form of assault.

## 2. Case Report

**Figure 1:**
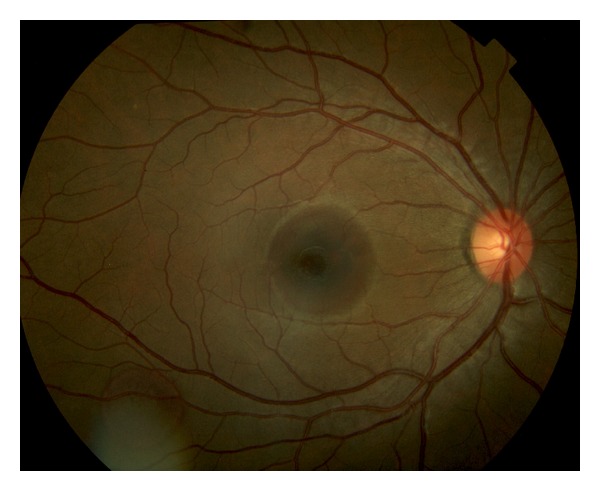
Two small localized subretinal hemorrhages in the right eye, one in the macula and another along the inferotemporal arcade, without any obvious choroidal rupture.

A 21-year-old white female was assaulted on New Year's Eve while walking with friends in downtown Denver. She was hit on the right side of her head and knocked out, and when she awoke a few minutes later, she noted decreased vision in the right eye. She was examined 3 days later. Her vision was 20/200 OD with normal anterior segments and normal intraocular pressures. Her right fundus exam revealed two subretinal hemorrhages, one in the macula and one along the inferotemporal arcade (Figure 1). There were no visible choroidal ruptures and no retinal detachment. The left fundus was normal.

## 3. Discussion

This patient sustained a submacular hemorrhage due to assault as part of the “knockout game.” While relatively mild, more serious injury is possible including ruptured globe, retinal detachment, or lens damage [2]. Although the injury and mechanism are not unique, the circumstances of this new form of assault are. Aside from more serious head and craniofacial injuries and even death, ocular injury may occur and even be overlooked due to other more serious nonocular injuries.

Shaken baby syndrome is a subset of head trauma that may bear some similarities to knockout game trauma [3]. The major difference, aside from the fact that shaken baby syndrome occurs in young children while the knockout game, thus far, has been limited to older teenagers and adults, is the mechanism of injury. The knockout game objective is to “knock out” the victim with a single punch to the head whereas shaken baby syndrome is characterized by shaking, repetitive acceleration-deceleration forces with or without blunt head impact. In addition, clinical signs of shaken baby syndrome include multiple and extensive retinal hemorrhages, traumatic retinoschisis, and perimacular folds, features typically not seen with a single punch to the head.

As this form of assault is becoming more frequent, it is important that first responders and emergency room personnel be aware of the potential for eye damage and inform patients of relevant ocular symptoms. Regardless of the motivations for the knockout game, such injuries are a new public health concern in urban environments.
